# Context-dependent interactions between variable-loop pulsed field ablation and cardiac implantable electronic devices: a controlled preclinical study

**DOI:** 10.1093/ehjopen/oeag124

**Published:** 2026-09-11

**Authors:** Masateru Takigawa, Ryosuke Kato, Iwanari Kawamura, Megumi Toda, Masaki Honda, Miho Negishi, Ryo Tateishi, Junji Yamaguchi, Kentaro Goto, Takuro Nishimura, Susumu Tao, Shinsuke Miyazaki, Tetsuo Sasano

**Affiliations:** Department of Cardiovascular Medicine, Institute of Science Tokyo, 1-5-45 Yushima, Bunkyo-ku, Tokyo 113-8519, Japan; Department of Cardiovascular Medicine, Institute of Science Tokyo, 1-5-45 Yushima, Bunkyo-ku, Tokyo 113-8519, Japan; Department of Cardiovascular Medicine, Akita University Graduate School of Medicine, Akita 010-0041, Japan; Department of Cardiovascular Medicine, Institute of Science Tokyo, 1-5-45 Yushima, Bunkyo-ku, Tokyo 113-8519, Japan; Department of Cardiovascular Medicine, Institute of Science Tokyo, 1-5-45 Yushima, Bunkyo-ku, Tokyo 113-8519, Japan; Department of Cardiovascular Medicine, Institute of Science Tokyo, 1-5-45 Yushima, Bunkyo-ku, Tokyo 113-8519, Japan; Department of Cardiovascular Medicine, Institute of Science Tokyo, 1-5-45 Yushima, Bunkyo-ku, Tokyo 113-8519, Japan; Department of Cardiovascular Medicine, Institute of Science Tokyo, 1-5-45 Yushima, Bunkyo-ku, Tokyo 113-8519, Japan; Department of Cardiovascular Medicine, Institute of Science Tokyo, 1-5-45 Yushima, Bunkyo-ku, Tokyo 113-8519, Japan; Department of Cardiovascular Medicine, Institute of Science Tokyo, 1-5-45 Yushima, Bunkyo-ku, Tokyo 113-8519, Japan; Department of Cardiovascular Medicine, Institute of Science Tokyo, 1-5-45 Yushima, Bunkyo-ku, Tokyo 113-8519, Japan; Department of Cardiovascular Medicine, Institute of Science Tokyo, 1-5-45 Yushima, Bunkyo-ku, Tokyo 113-8519, Japan; Department of Cardiovascular Medicine, Institute of Science Tokyo, 1-5-45 Yushima, Bunkyo-ku, Tokyo 113-8519, Japan; Department of Cardiovascular Medicine, Institute of Science Tokyo, 1-5-45 Yushima, Bunkyo-ku, Tokyo 113-8519, Japan

**Keywords:** Pulsed field ablation, Cardiac implantable electronic devices, Device–catheter interaction

## Abstract

**Aims:**

Pulsed field ablation (PFA) is increasingly used for atrial fibrillation and is being extended beyond pulmonary vein isolation to right atrial (RA) and superior vena cava (SVC) targets. As its use expands, interactions with cardiac implantable electronic devices (CIEDs) have become an important yet incompletely defined safety concern. We aimed to evaluate context-dependent interactions between PFA and diverse CIED systems in a controlled pre-clinical model.

**Methods and results:**

Thirteen swine underwent implantation of contemporary CIED systems. Seven animals received PFA across multiple device configurations, including dual-chamber pacemakers, transvenous ICD/CRT-D generator systems with single- or dual-coil ICD leads, a leadless ventricular pacemaker, and a subcutaneous ICD. Six control animals underwent catheter manipulation without energy delivery. PFA was performed in the left atrium (LA), RA, and SVC, including applications near transvenous leads and pre-specified delivery over exposed SVC defibrillator coils. Device parameters were assessed sequentially.

**Results:**

When performed at an anatomical distance from device components, LA-PFA was not associated with critical device dysfunction under the studied conditions, although minor parameter changes occurred in a minority of cases, particularly with septal atrial lead positioning. During RA/SVC procedures, parameter perturbations and atrial lead dislodgement were observed and were most consistent with catheter–lead mechanical interaction. PFA delivered overexposed ICD coils that produced localized electrical changes without evidence of acute global device failure. The leadless ventricular pacemaker and subcutaneous ICD maintained stable function.

**Conclusion:**

PFA did not cause catastrophic CIED dysfunction under the studied conditions; however, device-PFA interactions were strongly context-dependent, influenced by anatomical proximity, conductive exposure, and mechanical interaction.

## Graphical Abstract

**Graphical Abstract:**
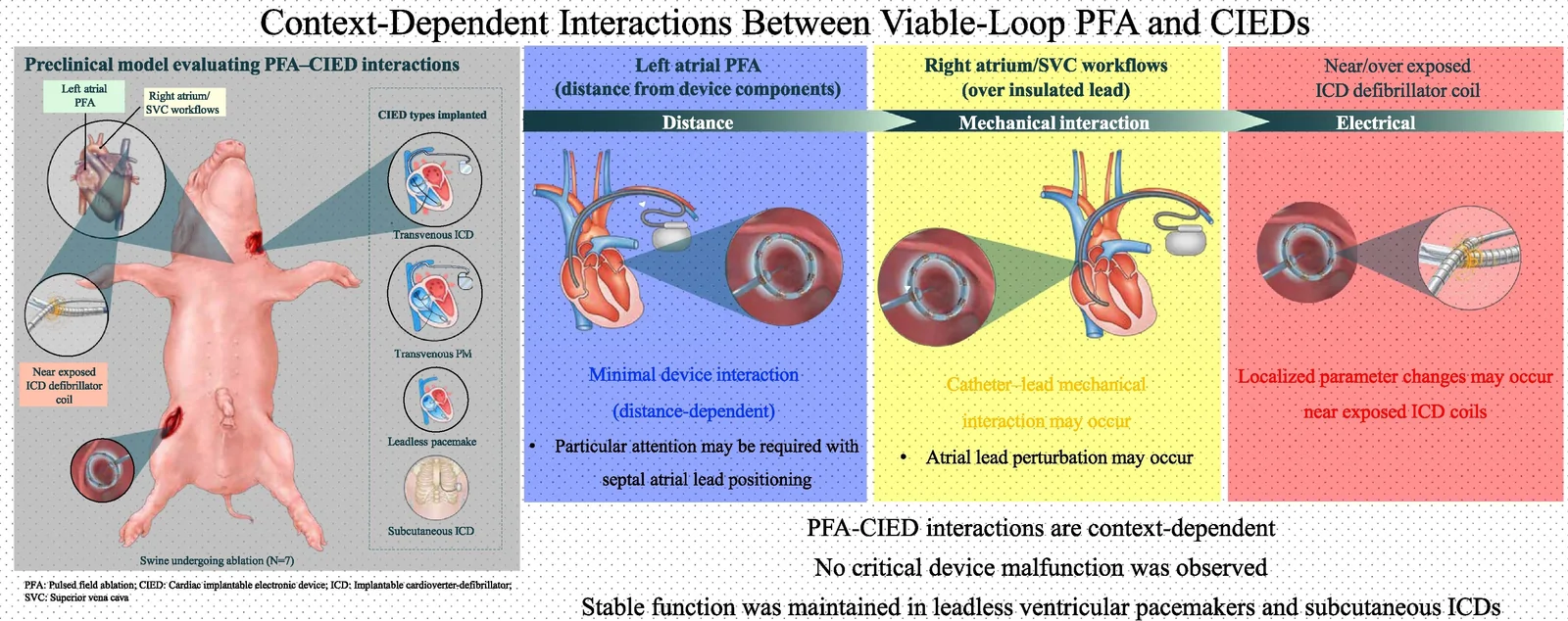

Translational perspective Pulsed field ablation (PFA) is increasingly used, yet its interaction with cardiac implantable electronic devices (CIEDs) remains incompletely defined. In this pre-clinical study, interactions between PFA and CIEDs were context-dependent. Left atrial PFA was not associated with overt device dysfunction under the studied conditions, whereas right atrial and superior vena cava procedures—particularly near-exposed defibrillator coils—may be associated with localized parameter changes and mechanical lead perturbation. These findings should be interpreted as hypothesis-generating and applied with caution in clinical settings. They underscore the potential for context-dependent device-PFA interactions and the need for careful catheter manipulation and device monitoring during right-sided PFA. Further studies in chronic and clinical settings are warranted to clarify long-term safety.

What’s New

- PFA was evaluated across diverse CIED configurations in a controlled swine model, including a catheter-manipulation control group.
- Interactions were context-dependent: left atrial PFA was largely uneventful, whereas right atrial/SVC procedures were associated with catheter–lead interactions.
- Applications of near-exposed ICD coils were associated with localized parameter changes without acute global device failure.
- The leadless ventricular pacemaker and subcutaneous ICD maintained stable function despite substantial PFA exposure.

## Introduction

Catheter ablation is an established therapy for AF, and pulsed field ablation (PFA) has emerged as a non-thermal modality that induces myocardial injury through irreversible electroporation, offering relative tissue selectivity compared with thermal energy sources.^1–3^ Early pre-clinical and clinical studies have demonstrated effective pulmonary vein isolation with a favourable safety profile, particularly regarding extracardiac structures.^4–8^ Consequently, PFA has been rapidly adopted in clinical practice.

With increasing PFA use, a growing proportion of patients undergoing ablation also carry cardiac implantable electronic devices (CIEDs), making device–PFA interactions an important safety consideration.^9^ Because PFA delivers high-voltage electrical energy, concerns remain regarding device interference, parameter changes, and interactions with intracardiac leads or defibrillator coils.

Available data suggest that PFA is generally compatible with contemporary CIED systems when confined to the left atrium (LA) and performed at a distance from device components.^10–13^ Transient electromagnetic interference has been reported, including noise detection, mode switching, and ventricular oversensing during energy delivery, typically without persistent malfunction.^14,15^ However, uncertainties remain. Prior studies have largely focused on left atrial workflows, with limited evaluation of right atrial (RA) and superior vena cava (SVC) settings, where transvenous leads frequently traverse the ablation field and exposed conductive components (e.g. defibrillator coils) may be encountered. Recent Europace commentary emphasized that evidence remains incomplete and that context-dependent risks require further clarification.^13^

Importantly, device interactions may not always be benign under extreme conditions. Device reset and damage have been reported following monopolar lattice-tip PFA,^16^ and permanent implantable cardioverter-defibrillator (ICD) damage has also been reported during left-sided septal ventricular tachycardia ablation using a lattice-tip catheter.^17^ In addition, a recent safety advisory described ventricular tachycardia/ventricular fibrillation events during radiofrequency delivery with the Sphere-9 lattice-tip catheter in patients with Biotronik ICD/CRT-D systems.^18^ Arcing has also been observed near metallic intracardiac structures.^19^ Takahashi et al. reported irreversible internal circuit damage of an intracardiac defibrillation/DC system associated with VARIPULSE™ under extreme proximity to conductive components.^20^ These findings suggest that compatibility between PFA/ablation systems and CIEDs is not universal but depends on factors such as anatomical distance, device architecture, conductive exposure, energy mode, and procedural mechanics.

The VARIPULSE system incorporates a catheter design that may influence mechanical interactions with transvenous leads and local electric field distribution compared with fixed-geometry or spline-based systems. However, systematic evaluation across diverse CIED types—including transvenous pacemakers, ICDs, leadless pacemakers, and subcutaneous ICDs (S-ICDs)—remains limited, particularly in RA/SVC and coil-proximity settings.

Accordingly, this pre-clinical study was designed to assess the effects of PFA on CIED function in a controlled swine model. By evaluating device performance during LA ablation, RA/SVC ablation, and applications over leads and defibrillator coils, we aimed to define the electrical and mechanical safety profile of this system across diverse device configurations.

## Methods

### Experimental animal model

Thirteen female swine (age 3.3–3.5 months; weight 55.9–56.5 kg) were pre-medicated intramuscularly with midazolam 0.25 mg/kg, butorphanol tartrate 0.125 mg/kg, and medetomidine hydrochloride 25 µg/kg. After intravenous thiamylal 5 mg/kg, the animals were intubated, and anaesthesia was maintained with 2–3% isoflurane. Ventilation was provided using a Prima SP Anaesthesia System (Penlon) with room air supplemented with oxygen. A jugular venous sheath was placed for drug and fluid administration. Amiodarone (7–13 mg/kg) was infused to prevent ventricular arrhythmias, and norepinephrine was titrated as needed for hypotension. Core temperature, arterial blood pressure, and oxygenation were continuously monitored.

### Device implantation

As shown in *Table 1*, CIEDs were implanted in 13 swine. Devices with > 5 years battery longevity and normal function were used, and all leads were new. All CIED implantation, lead fixation, catheter manipulation, and PFA delivery were performed during the same acute experimental session. Therefore, all transvenous leads were newly implanted and had not undergone chronic lead maturation, tissue ingrowth, or fibrotic encapsulation before catheter manipulation or PFA delivery.

**Table 1:** Implanted devices and procedure details

(A) Implanted CIED configurations in the study swine					
	PM, A-lead	PM, RV-lead	ICD, V-lead	Leadless V	S-ICD
**PFA (+)**					
Swine 1	A1	V1	no	no	no
Swine 2	A2	V2	no	no	no
Swine 3	A3	V3	no	no	no
Swine 4	A4	no	V4 (Single-coil)	no	no
Swine 5	A5	no	V5 (Dual-coil)	no	no
Swine 6	no	no	V6 (Dual-coil)	yes	no
Swine 7	no	no	V7 (Dual-coil)	no	yes

	PM, A-lead	PM, RV-lead	ICD, V-lead	Leadless V	S-ICD
**PFA (−)**					
Swine 8	rA1	no	no	no	no
Swine 9	rA2	no	no	no	no
Swine 10	rA3	no	no	no	no
Swine 11	rA4	no	no	no	no
Swine 12	rA5	no	no	no	no
Swine 13	rA6	no	no	no	no

(B) Lead characteristics: product name and manufacturer		
Lead name	Product name	Manufacturer
A1	INGEVITY™+ (pacing lead)	Boston Scientific
A2	CapSure Sense MRI™ SureScan™ (pacing lead)	Medtronic
A3	Tendril™ STS (pacing lead)	Abbott
A4	CapSureFix Novus MRI™ SureScan™ (pacing lead)	Medtronic
A5	Tendril™ STS (pacing lead)	Abbott
V1	INGEVITY™+ (pacing lead)	Boston Scientific
V2	CapSureFix Novus MRI™ SureScan™ (pacing lead)	Medtronic
V3	Tendril™ STS (pacing lead)	Abbott
V4	Sprint Quattro Secure S MRI™ SureScan™ (ICD lead)	Medtronic
V5	DURATA™ ICD Lead (dual-coil ICD lead)	Abbott
V6	Sprint Quattro™ (dual-coil ICD lead)	Medtronic
V7	RELIANCE™ (dual-coil ICD lead)	Boston Scientific
rA1	INGEVITY™+ (pacing lead)	Boston Scientific
rA2	CapSureFix Novus MRI™ (pacing lead)	Medtronic
rA3	Tendril™ STS (pacing lead)	Abbott
rA4	CapSureFix Novus MRI™ (pacing lead)	Medtronic
rA5	INGEVITY™+ (pacing lead)	Boston Scientific
rA6	Tendril™ STS (pacing lead)	Abbott

Seven swine (Swine 1–7) underwent PFA delivery (‘PFA group’), whereas six (Swine 8–13) served as controls and underwent catheter manipulation without energy delivery.

#### PFA group configurations

- Swine 1–3: Dual-chamber pacemaker
- Swine 4: Dual-chamber ICD system with a single-coil transvenous ICD lead
- Swine 5: Dual-chamber ICD system with a dual-coil transvenous ICD lead
- Swine 6: CRT-D generator connected to a transvenous dual-coil ICD lead without atrial or LV lead implantation, together with a leadless ventricular pacemaker
- Swine 7: S-ICD system and CRT-D generator connected to a transvenous dual-coil ICD lead, without atrial or LV lead implantation

#### Control group

- Swine 8–13: Single atrial lead to minimize invasiveness while enabling assessment of catheter–lead interaction during LA/RA/SVC navigation

**Figure 1:**
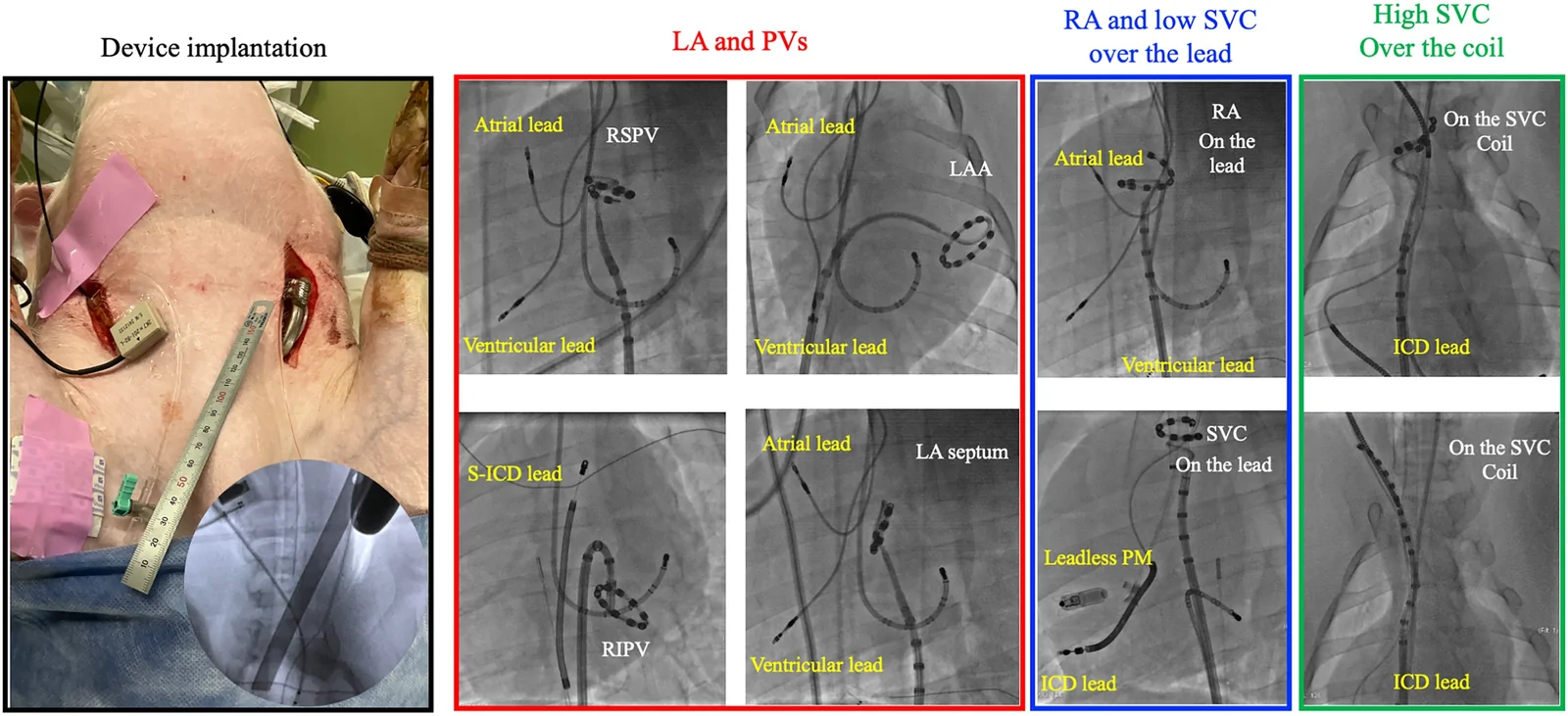
Procedural overview of pulsed field ablation relative to cardiac implantable electronic devices. Demonstration of the cardiac device implantation to Swine and representative fluoroscopic images illustrating the procedural and anatomical contexts of PFA delivery in relation to implanted device components, including the pulmonary veins and left atrium, electrically insulated lead segments traversing the right atrium and low superior vena cava, and exposed defibrillator coils within the high superior vena cava.

All procedures were performed under general anaesthesia. Transvenous devices were implanted via the jugular vein, with leads advanced to the RA and right ventricle (RV) under fluoroscopic guidance. After confirming acceptable pacing and sensing parameters, leads were secured and the pulse generator was placed in a cervical subcutaneous pocket (∼15 cm from the SVC; *Figure 1*). Lead position was confirmed in at least two projections at implantation and at each reassessment timepoint.

The leadless pacemaker was implanted via a transjugular approach. A steerable delivery catheter was advanced to the RV, and after confirming appropriate septal positioning with acceptable parameters, the device was deployed and fixated using nitinol tines. Stable fixation was confirmed before release.

The S-ICD was implanted using a standard three-incision technique (parasternal, xiphoid, and lateral chest wall). A subcutaneous pocket was created laterally, and the lead was tunnelled parallel to the sternum with electrodes positioned according to anatomical landmarks. The generator was placed in the pocket and incisions were closed.

### Device programming and peri-procedural management

CIEDs were interrogated at baseline and at pre-specified timepoints using manufacturer-specific programmers. Pacemakers were programmed to DDD mode (lower rate 70–80 bpm). All sensing and detection parameters were maintained at nominal manufacturer settings.

ICD shock therapy was disabled during catheter manipulation and PFA delivery and was re-enabled at the end of the procedure to confirm appropriate detection and therapy for ventricular fibrillation.

Intracardiac EGMs were monitored in real time but not systematically archived; analyses focused on pre- and post-delivery device parameters.

### Mapping protocol

A decapolar catheter was inserted through the right or left internal jugular vein and positioned in the coronary sinus. The left and right atria were mapped during CS pacing, with a mapping catheter (OPTRELL, Biosense Webster, Inc.) and three-dimensional activation mapping (CARTO 3 system, Biosense Webster, Irvine, CA, USA). For consistency, mapping was performed at a stable cycle length, and catheter positions were recorded at each stage to support comparisons of signal disturbances and lead interactions.

### PFA delivery protocol

Pulsed-field ablation was performed using a fully integrated system consisting of a circular catheter (VARIPULSE), a generator (TRUPULSE), and an irrigation pump (nGEN). Each application consisted of biphasic bipolar pulses (1800V, 250 ms). A standard delivery comprised three consecutive applications automatically delivered with a 10 s pause. Between deliveries, the catheter was slightly rotated or repositioned to mimic clinical manipulation and avoid repeated energy delivery at an identical interface.

In clinical practice, pulmonary vein isolation (PVI) with the VARIPULSE system is typically performed using four deliveries (12 applications) per PV, corresponding to 16 deliveries (48 applications) for four veins.

Because porcine pulmonary venous anatomy typically consists of two left atrial ostia—a right superior PV and an inferior/common PV trunk with bifurcating branches—ablation was performed at distal and proximal antral levels for each accessible pulmonary venous structure. At each level, four deliveries were applied, resulting in eight deliveries per venous structure (24 applications) and 16 deliveries (48 applications) in total for PVI. Additional LA ablation was performed to simulate adjunctive lesion sets used in clinical AF ablation, including posterior wall isolation (roof and floor lines, five deliveries each) and a lateral LA line (six deliveries), corresponding to 16 additional deliveries (48 applications). Thus, the overall LA workflow consisted of °C 32 deliveries (96 applications), reflecting combined PVI and linear lesion sets.

During RA and SVC ablation, energy delivery was guided by anatomical landmarks without intentional targeting of transvenous leads. However, because right-sided leads traverse the SVC–RA junction, SVC ablation was inherently performed with the catheter crossing the lead trajectory, confirmed in two fluoroscopic projections. At least five deliveries were applied within the SVC, with up to 15 across the entire segment depending on catheter position. Limited ablation sometimes extended into the lower RA.

In swine 5–7, at least 10 deliveries were performed in proximity to, and occasionally directly over, exposed SVC defibrillator coils as a pre-specified worst-case test rather than clinical practice. Due to anatomical constraints, all SVC coils were located in the high SVC region.

In swine 8–13, catheter manipulation was performed in the LA and RA without energy delivery to assess the mechanical impact on lead parameters.

### Safety assessment

Device parameters were assessed at pre-defined procedural stages: before and after LA ablation, before and after RA/SVC ablation over transvenous leads, and before and after ablation over the SVC shock coil. At each stage, sensing amplitude, pacing threshold (0.4 ms), lead impedance, and shock impedance were recorded, with measurements repeated after a stabilization period ( ≥ 5 min).

Device interrogation was performed before and after each stage to evaluate parameter stability and screen for critical device malfunction, including power-on reset, electrical reset, transition to backup pacing mode, sudden battery loss, and loss of telemetry. Event logs were not systematically recorded.

Pre-specified safety endpoints included the following:

- Lead dislodgement, defined as fluoroscopic evidence of lead position change associated with loss of capture;
- Sustained parameter shift, defined as a ≥ 0.5 V increase in pacing threshold, a ≥ 30% reduction in P- or R-wave amplitude, or a ≥ 10% change in lead impedance compared with baseline;
- Out-of-range sensing or impedance values, defined as P-wave amplitude < 1 mV (atrial leads), R-wave amplitude < 4 mV (ventricular leads), or lead impedance < 300 Ω or > 1500 Ω;
- Critical device malfunction, including power-on reset, electrical reset, transition to backup pacing mode, sudden battery depletion, or loss of telemetry.

Sustained abnormalities were defined as those persisting at repeat interrogation after stabilization and at final interrogation at the end of the procedure.

In animals with ICD systems, arrhythmia detection and therapy delivery were assessed as a separate endpoint. Ventricular fibrillation was induced at the end of the experiment to confirm appropriate detection and shock delivery.

### Statistical analysis

Continuous paired data are presented as median (interquartile range) and were compared using the Wilcoxon signed-rank test. A two-sided *P*-value < 0.05 was considered statistically significant. All analyses were performed with JMP software, version 18.2.0 (SAS Institute, Cary, NC).

### Ethical approval

The study protocol of the swine experiment received approval from the Institutional Animal Care and Use Committee (IACUC) of Johnson & Johnson K.K. (JJKK227_20240916).

## Results

### Study population and device configuration

Five swine had both atrial and ventricular transvenous leads (three pacemaker and two ICD systems), whereas two additional swine had no atrial transvenous lead [one with a leadless pacemaker plus a transvenous ICD lead, and one with a subcutaneous ICD (S-ICD) plus a transvenous ICD lead], as described in *Table 1*. Details of PFA delivery in the intervention group are summarized in Supplementary material online, *Table S1*. In the control group (*n* = 6), atrial pacing leads were implanted and catheter manipulation was performed without PFA delivery.

### Overall safety outcomes

Critical device malfunction—including power-on reset, electrical reset, transition to backup pacing mode, sudden battery loss, and loss of telemetry—was not observed during the experiment. Although detailed device event logs were not systematically analysed, no clinically relevant alerts or automatically flagged diagnostic abnormalities were identified during post-procedural interrogation. Safety endpoints related to lead parameters are described in the following section.

### PFA in the left atrium

**Figure 2:**
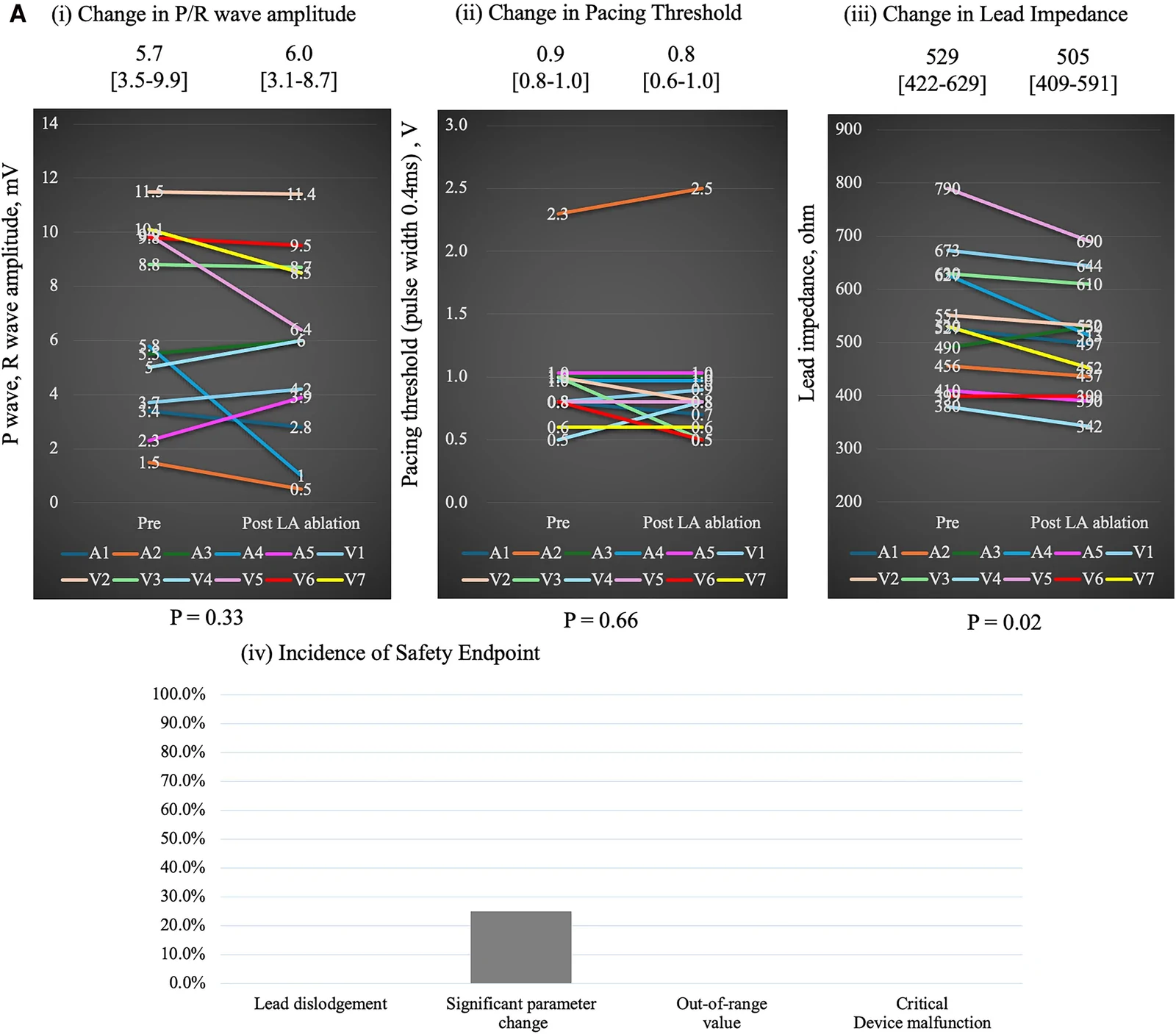

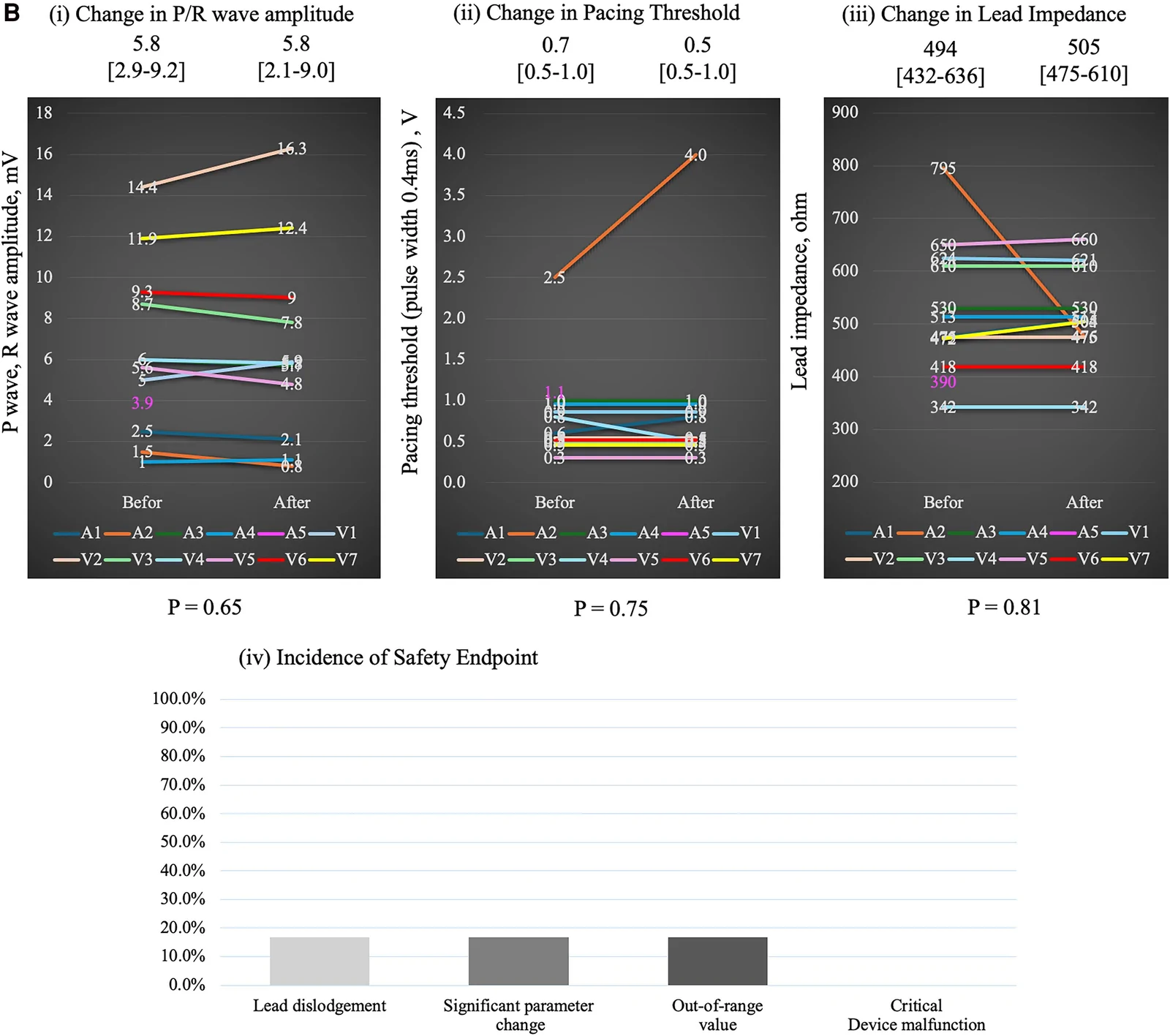

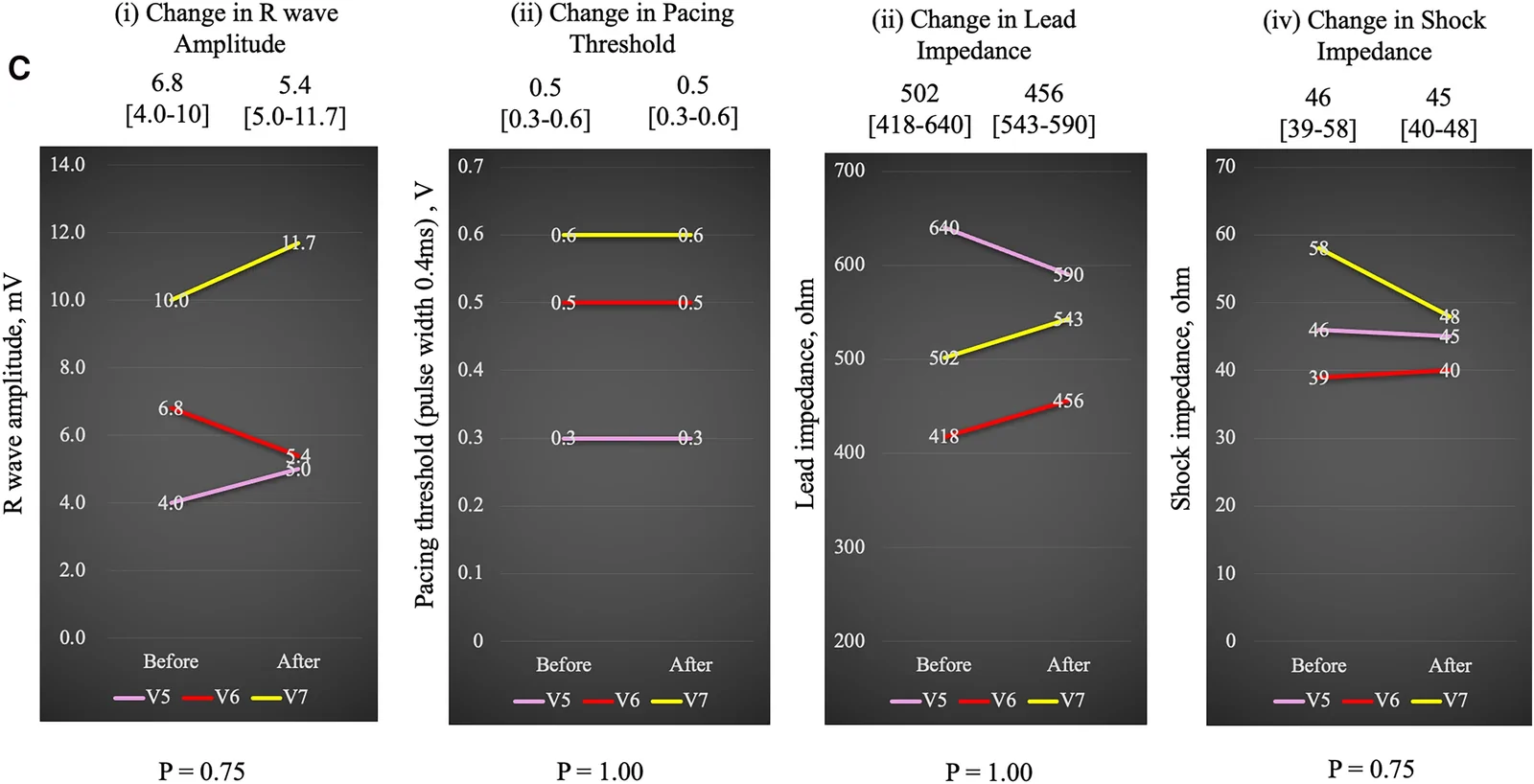
Parameter changes during PFA and incidence of safety endpoint. (*A*) Before and After PFA in LA (*B*) Before and After PFA in RA, SVC (*C*) Before and After PFA near/over the SVC-Coil. (*A*) Before and after left atrial (LA) PFA. (*B*) Before and after right atrial/superior vena cava isolation (RA/SVC) procedures. (*C*) Before and after pre-specified worst-case PFA applications delivered directly over exposed SVC defibrillator coils (‘SVC-coil’). Individual animal values are shown [bars indicate median (25%ile—75%ile)]. Parameters assessed included sensing amplitude (mV), pacing threshold (V), and lead/shock impedance (Ω), as applicable to each device type. Measurements were obtained immediately before and after each stage; missing values reflect inability to measure due to lead dislodgement or procedural constraints.

**Figure 3:**
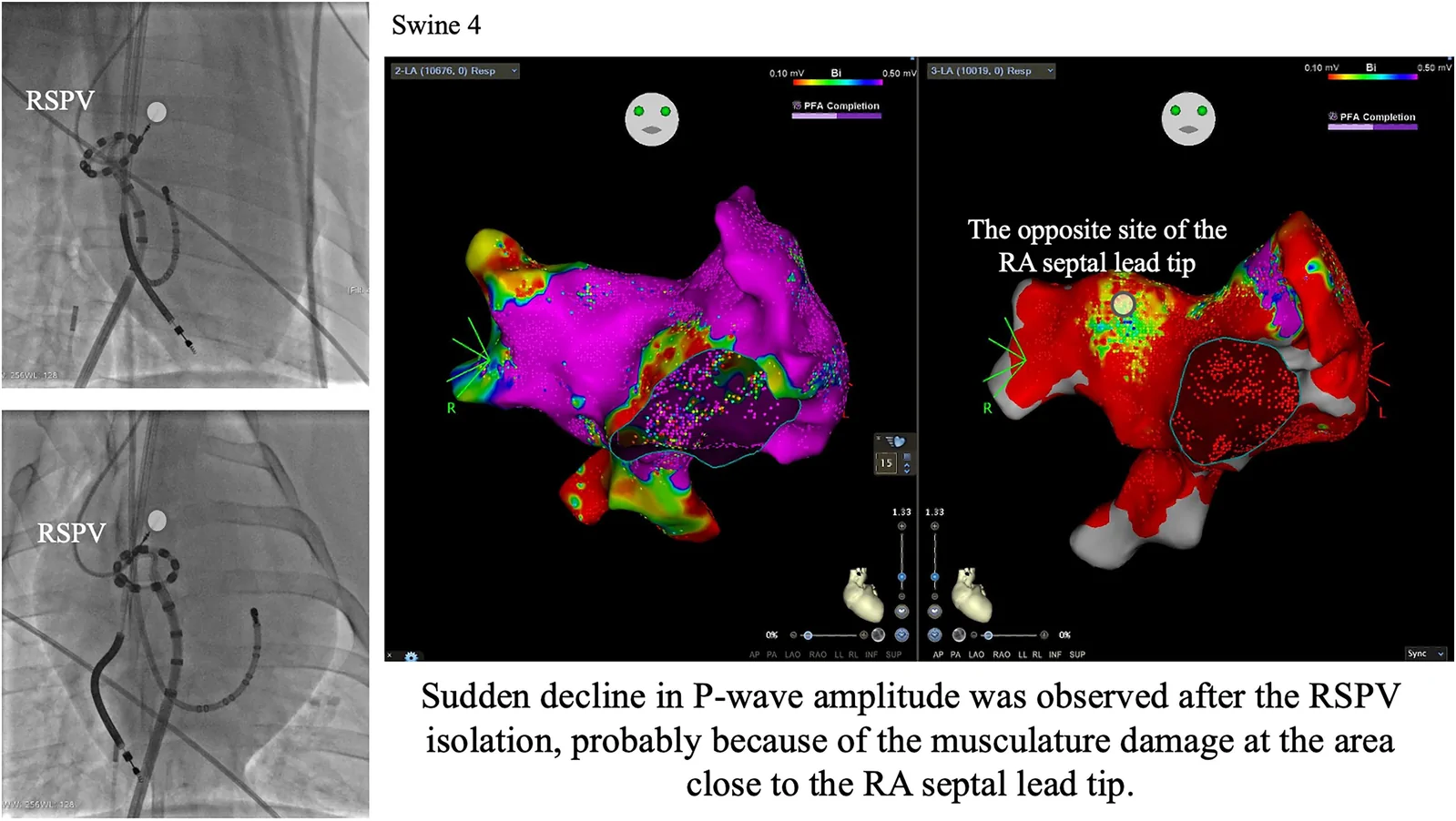
Representative case demonstrating acute reduction in atrial lead P-wave amplitude following right superior pulmonary vein PFA. RSPV, right superior pulmonary vein.

A total of 98 (86–120) applications were delivered in the LA, including a median of 50 (49–53) for PVI, with the remainder for additional LA ablation across all seven swine. As shown in *Figure 2A*, atrial and ventricular sensing amplitudes and pacing thresholds were not significantly altered during LA PFA. Lead impedance decreased from 529 (422–629) Ω to 505 (409–591) Ω (6% reduction; *P* = 0.02), with a similar decrease observed in the control group. No critical device malfunction occurred; however, parameter changes were detected in three swine (atrial leads in Swine 2 and Swine 4, and a ventricular lead in Swine 5). In Swine 2 and Swine 4, atrial leads (A2 and A4) were positioned on the septal side due to anatomical difficulty with right atrial appendage placement. In these animals, atrial sensing amplitude and lead impedance decreased after right superior pulmonary vein (RSPV) isolation. In Swine 4, left atrial voltage mapping demonstrated the extension of a low-voltage area to the contralateral side of the atrial lead implantation site following RSPV PFA (*Figure 3*). In Swine 5, sensing amplitude and impedance of the ventricular lead (V5) decreased after LA PFA but remained sufficiently within normal ranges until the end of the procedure.

During LA applications, a single transient noise was observed on real-time intracardiac electrograms during PFA delivery (see Supplementary material online, *Figure S1*), without persistent abnormalities.

### PFA in the right atrium and superior vena cava

In the RA and SVC, a median of 44 (24–57) applications were delivered. In SVC, atrial and ventricular leads anatomically traversed the ablation field.

As shown in *Figure 2B*, atrial sensing amplitude, pacing threshold, and impedance were not significantly altered during RA/SVC PFA in most animals. Ventricular lead parameters likewise remained stable. Although no critical device malfunction was observed, parameter changes occurred in two swine. In Swine 5, the atrial lead (A5) was completely dislodged during VARIPULSE catheter manipulation in the SVC, most consistent with mechanical interaction. In Swine 2, the atrial lead (A2) was positioned suboptimally, with low baseline sensing amplitude and elevated pacing threshold. Although not dislodged, a slight change in lead tip angle was observed. During SVC ablation, atrial sensing amplitude, pacing threshold, and impedance deteriorated while catheter manipulation and PFA delivery were interleaved. Because device interrogation was performed intermittently after manipulation and multiple applications, the timing of these changes (manipulation vs. PFA) could not be determined. Notably, these changes were confined to atrial leads, with ventricular leads remaining stable. Similar perturbations were also observed during catheter manipulation without PFA in controls, supporting a role for catheter–lead mechanical interaction.

In Swine 4, parameters of the single-coil ICD lead (V4)—including sensing amplitude, pacing threshold, lead impedance, and shock impedance—remained stable before and after SVC PFA delivered over an electrically insulated lead segment.

### PFA delivered over the ICD coil

**Figure 4:**
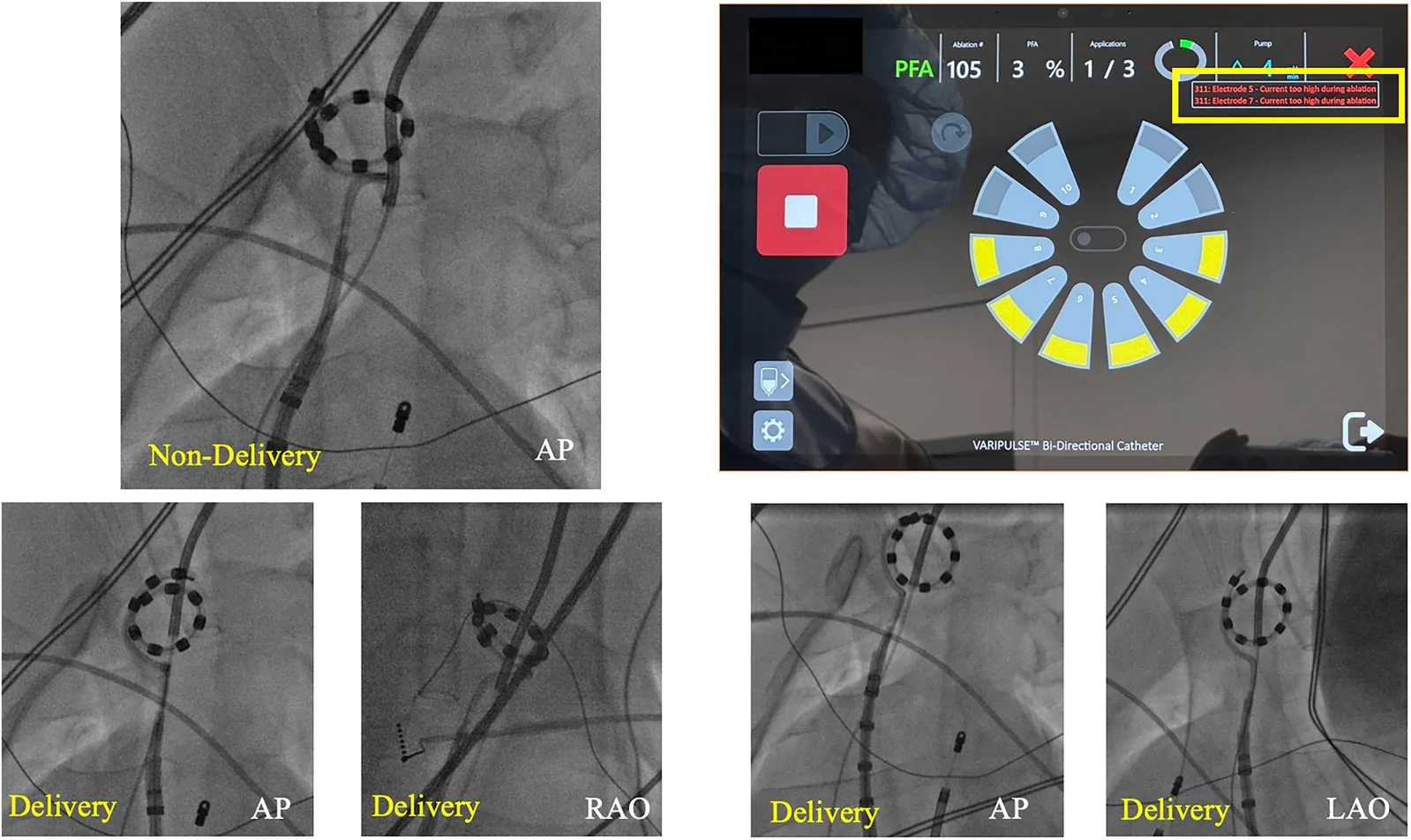
Examples of PFA delivery behaviour during applications near the superior vena cava (SVC) defibrillator coil. “Delivery” indicates that the programmed PFA pulse sequence was completed without system interruption, whereas “Non-delivery” indicates that the pulse sequence was initiated but automatically terminated before completion because excessive current was detected. The upper-right panel shows the generator display during a non-delivery event, with “current too high during ablation” warnings for catheter electrodes 5 and 7. The fluoroscopic examples demonstrate that apparent catheter-coil proximity or overlap did not invariably result in automatic interruption of PFA delivery. AP, anteroposterior; LAO, left anterior oblique; RAO, right anterior oblique.

In three swine with dual-coil ICD leads, a median of 57 (37–71) PFA applications were delivered over the SVC coil (*Figure 2C*). In one animal (Swine 7), shock impedance decreased markedly from 58 to 48 Ω, without changes in R-wave sensing, RV pacing threshold, or ventricular impedance. Although the clinical significance remains uncertain, this reduction met the pre-defined safety endpoint. When the catheter electrode was clearly not overlapping the SVC coil on fluoroscopy, PFA delivery was not interrupted. However, PFA delivery was not consistently terminated even when catheter–coil contact was suspected (*Figure 4*).

### PFA and ventricular leadless pacemaker

In Swine 6, a leadless ventricular pacemaker was implanted in addition to the ICD lead. Even after 200 applications were delivered in the RA and LA, R-wave sensing, pacing threshold, and impedance were not affected. During PFA applications, a single noise event was observed in the real-time intracardiac electrograms (see Supplementary material online, *Figure S2*).

### PFA and subcutaneous ICD

**Figure 5:**
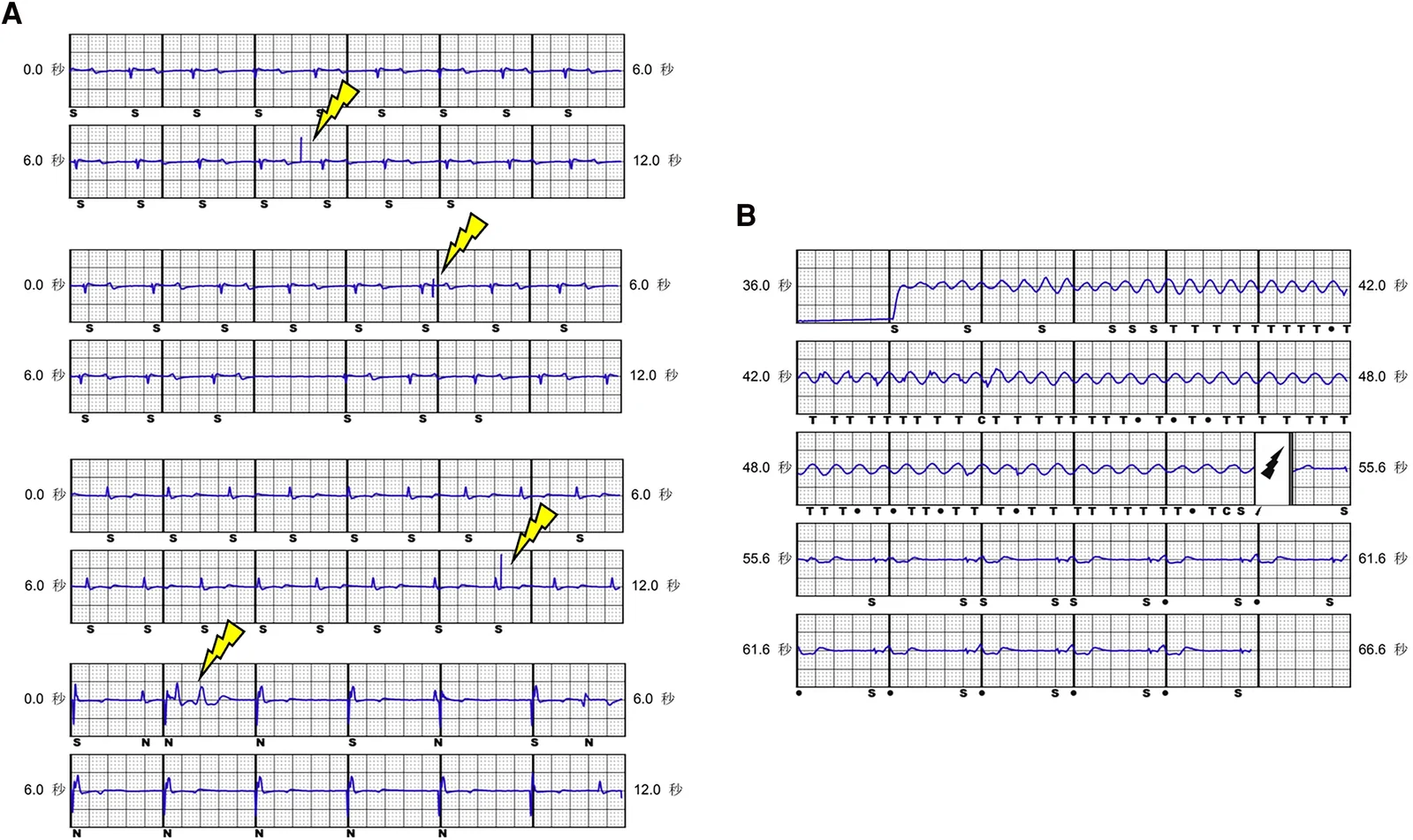
Intracardiac electrograms recorded by the S-ICD during PFA and subsequent defibrillation. ‘Over-coil’ was defined as fluoroscopic overlap between the ablation electrodes and the SVC defibrillator coil in at least two orthogonal projections with stable catheter position, whereas ‘near-coil’ indicated close adjacency without overlap. In near-coil configurations, energy delivery was generally feasible, whereas in over-coil configurations, PFA applications were frequently (but not always) interrupted or terminated prematurely. (*A*) Interference detected by the defibrillator during PFA (*B*) Preserved defibrillator function.

At implantation, appropriate electrograms were recorded only in the primary sensing vector, with a shock impedance of 55 Ω. Despite 188 PFA applications, sensing remained appropriate in both primary and alternate vectors, and shock impedance was maintained at 50 Ω. During applications, the S-ICD detected a small single spike as noise (*Figure 5A*). Ventricular fibrillation was manually induced, and the ICD appropriately sensed and terminated the arrhythmia (*Figure 5B*).

### Defibrillation function

At the end of the procedure, ventricular fibrillation was induced in Swine 4, 5, and 7 and was appropriately detected and terminated by shock therapy. Notably, in Swine 7—despite a marked reduction in shock impedance after PFA—defibrillation function remained intact.

### Catheter manipulation without PFA delivery

**Figure 6:**
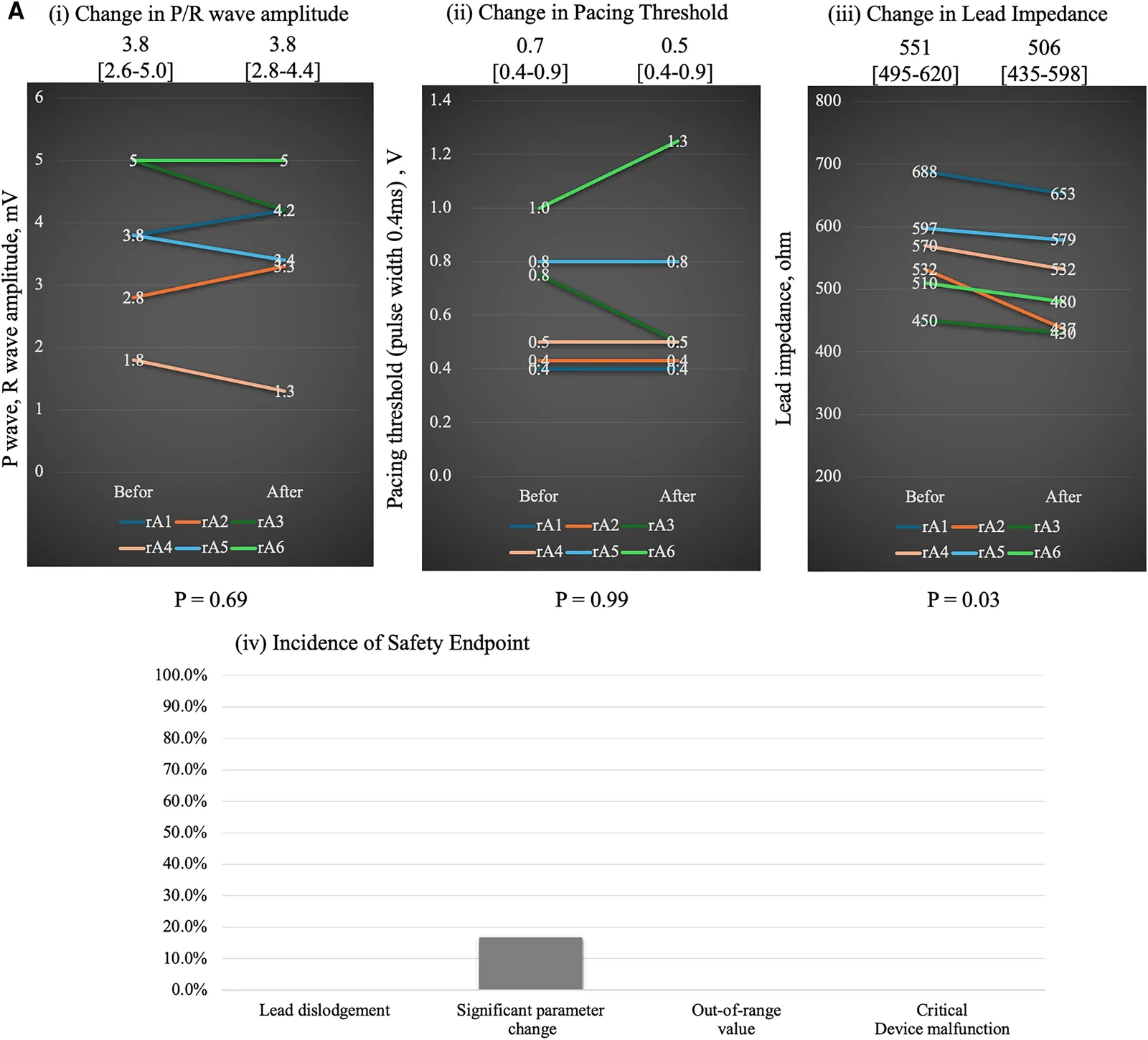

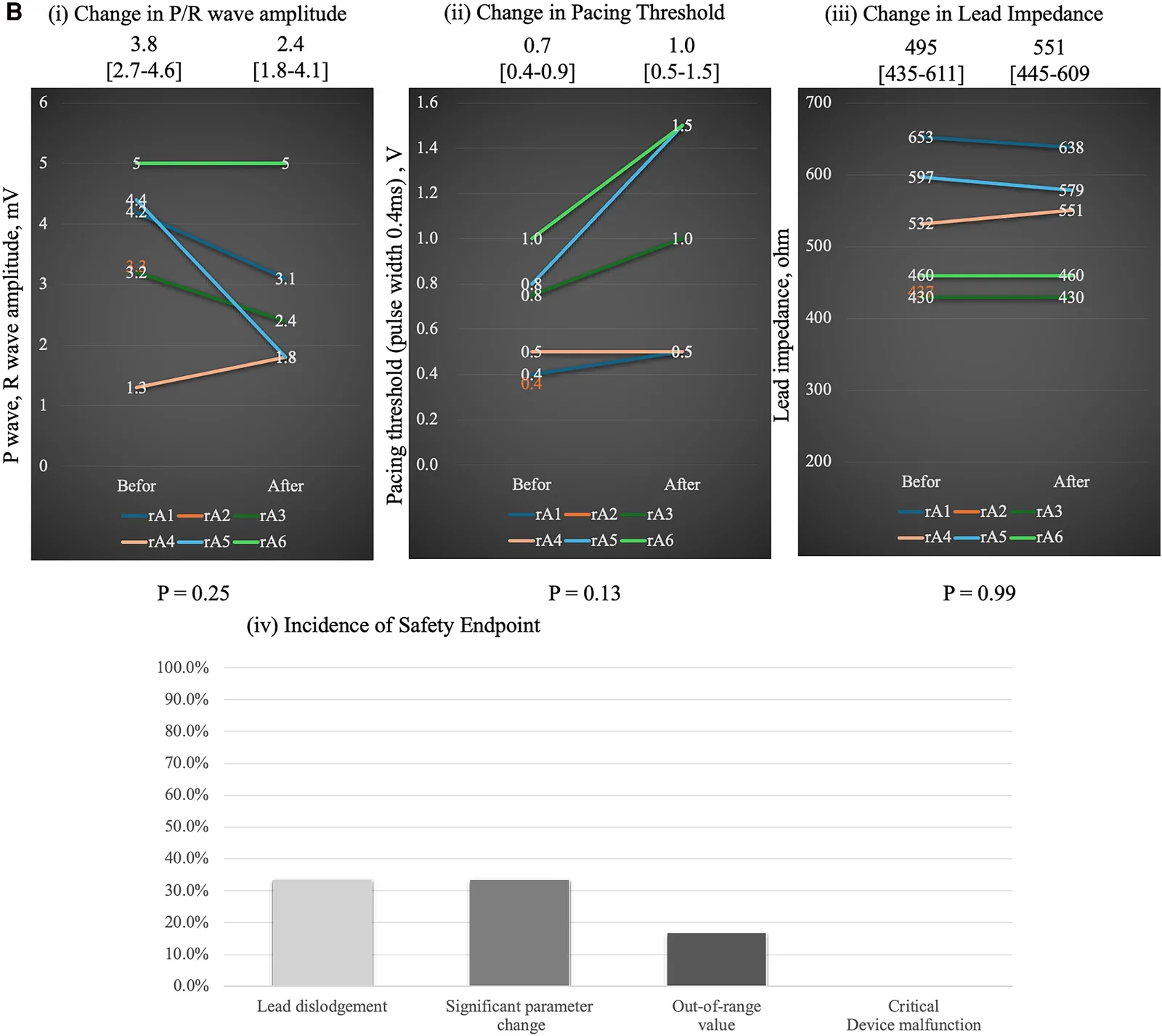
Parameter changes during catheter manipulation without PFA. (*A*) Before and After LA catheter manipulation (*B*) Before and After RA, SVC catheter manipulation.

To assess the isolated effect of catheter manipulation, the VARIPULSE catheter was manipulated in the LA and RA without PFA in six swine. As shown in *Figure 6*, no apparent impact was observed in most cases. However, complete atrial lead dislodgement occurred in one swine (Swine rA2) and micro-dislodgement in another (Swine rA5) during SVC manipulation over atrial and ventricular leads (*Figure 6*), without critical device malfunction.

## Discussion

### Overview of major findings

The present study provides a pre-clinical evaluation of context-dependent interactions between PFA and CIEDs, with particular focus on right-sided procedural settings.

The principal findings are as follows:

- When performed at an anatomical distance from device components, LA PFA was not associated with critical device malfunction in this acute experimental setting.
- During RA and SVC procedures, acute parameter changes were observed in a subset of cases, most consistent with catheter–lead mechanical interaction. In addition, complete atrial lead dislodgement in one swine and atrial lead micro-dislodgement in another occurred during SVC catheter manipulation near transvenous leads, without evidence of critical device malfunction.
- PFA delivered near-exposed ICD coils was associated with localized parameter changes without evidence of acute global device failure, although the clinical significance remains uncertain.
- Leadless pacemakers and subcutaneous ICDs maintained stable sensing and therapy function despite extensive PFA exposure.

### Electrical compatibility of PFA with CIEDs: consistency with prior evidence

Prior clinical and pre-clinical studies have suggested that PFA is broadly compatible with contemporary CIED platforms.^10–13^ The findings from our acute swine model are consistent with these observations. Under the present experimental conditions, PFA delivered using the VARIPULSE catheter did not result in overt or critical device dysfunction. However, this interpretation should be tempered by the occurrence of acute parameter changes in a limited number of cases.

A modest but statistically significant reduction in lead impedance was observed following LA PFA. However, this finding likely reflects, at least in part, time-dependent post-implantation effects rather than a purely PFA-specific interaction, as a similar decrease was also observed in control animals undergoing catheter manipulation without energy delivery.

Notably, in two animals with atrial leads positioned in the right atrial septum, a reduction in *P*-wave amplitude was observed after RSPV PFA, and electroanatomical mapping demonstrated voltage attenuation in the contralateral left atrial region.

Given the single-shot nature of the VARIPULSE system and the relatively large catheter size compared with the small-animal LA, adjacent atrial tissue may be exposed to broader electric fields beyond the immediate electrode interface, or the tissue–lead interface angle may be altered by mechanical catheter movement. Accordingly, when atrial leads are positioned near the interatrial septum or Bachmann’s bundle region in clinical settings, unintended myocardial effects cannot be excluded. These observations indicate that even during LA ablation, device–tissue interactions may occur in a context-specific manner.

Otherwise, no clinically significant deterioration in sensing amplitude, pacing threshold, or lead impedance was observed following LA PFA. These findings suggest that PFA delivered at an anatomical distance from the lead system is unlikely to cause sustained device dysfunction under the present conditions. However, given the limited sample size and the occurrence of parameter changes in a small number of cases, these findings should be interpreted with caution.

### Procedural considerations during right atrial and SVC ablation

In contrast to LA procedures, RA and SVC workflows were associated with sporadic device parameter changes observed during both PFA delivery and catheter manipulation, suggesting a contribution of catheter–lead interaction. In the SVC, transvenous leads traverse the ablation field, and catheter–lead contact is often unavoidable. Despite this, no consistent pattern of electrical interference was observed across most leads, supporting the interpretation that PFA delivered over or adjacent to insulated lead segments is unlikely to produce major electrical effects under the studied conditions.

Additional observations support a mechanical contribution. A similar or higher incidence of parameter changes and lead dislodgements was observed in control animals without PFA delivery (*Figure 2B* and *Figure 6B*), indicating that catheter manipulation alone may influence lead position and contact. Furthermore, changes were confined to atrial leads, whereas ventricular leads remained stable, likely reflecting differences in lead slack and anchoring. Ventricular leads are typically positioned with sufficient slack across the tricuspid valve and within the right ventricle, which may reduce their susceptibility to rotational or traction forces generated during catheter manipulation. Fluoroscopic observations also suggested subtle positional changes in atrial lead tips during catheter manipulation.

A clinically relevant example of atrial lead parameter instability was observed in Swine 2. The atrial lead A2 was initially positioned suboptimally, with low baseline sensing amplitude and an elevated pacing threshold, indicating limited sensing and capture reserve. During the SVC workflow, atrial sensing amplitude, pacing threshold, and lead impedance progressively deteriorated in the setting of repeated catheter manipulation and PFA delivery near the atrial lead. Taken together, we consider mechanical lead–tissue or catheter–lead interaction, rather than direct PFA-induced lead injury, to be the most plausible primary mechanism. Nevertheless, because this occurred during right-sided PFA near the atrial lead, the finding remains clinically relevant and supports heightened caution and repeated lead interrogation during RA/SVC PFA, particularly in patients with marginal baseline lead parameters or vulnerable lead positions.

Complete atrial lead dislodgement in one swine and atrial lead micro-dislodgement in another further highlight that SVC catheter manipulation using the VARIPULSE system may represent a clinically relevant procedural hazard near transvenous CIED leads. These events occurred during manipulation close to atrial and ventricular leads and were not accompanied by critical device malfunction. However, lead displacement may still result in loss of capture, sensing abnormalities, or the need for lead revision in clinical practice. Therefore, catheter–lead mechanical interaction is the most plausible principal mechanism of parameter perturbation and lead displacement during RA/SVC workflows.

These findings should be interpreted cautiously. All leads were newly implanted, and the smaller SVC/RA dimensions in swine may have increased susceptibility to mechanical lead displacement. Thus, these observations should be viewed as signals of procedural mechanical vulnerability under sensitive experimental conditions, rather than direct estimates of clinical dislodgement risk during human PFA procedures. Although a direct electrical contribution cannot be entirely excluded, careful catheter manipulation and device interrogation may be warranted during right-sided PFA near transvenous leads.

### Device- and lead-specific considerations

Device- and lead-specific factors may modulate susceptibility to PFA-related interactions. Differences in sensing circuitry, filtering algorithms and tolerance to transient voltage perturbations may influence device behaviour. Lead characteristics, including insulation properties and geometry, may also affect both the mechanical behaviour during catheter manipulation and the local electric field distribution.

In particular, integrated bipolar ICD leads to incorporate the defibrillation coil into the sensing circuit, resulting in a longer sensing vector compared with dedicated bipolar leads. This lead configuration was represented among the ICD leads used in the present study and may theoretically increase susceptibility to electrical perturbation under certain procedural conditions. However, because lead configuration-specific susceptibility was not systematically evaluated, these considerations remain speculative. Because the biological effects of PFA depend on waveform characteristics, electrode configuration, and energy-delivery parameters, the present findings should be interpreted in the context of the specific PFA system used in this study (VARIPULSE) and may not be directly generalizable to other PFA platforms with different waveforms or electrode geometries. For example, severe electromagnetic interference, including device reset, premature battery depletion, and device malfunction, has been reported in association with monopolar PFA systems in certain procedural contexts.^16^ Accordingly, interactions between PFA and CIED systems are likely to be context-dependent rather than uniform across platforms.

### PFA near-exposed conductive components (ICD coils): potential mechanisms and unresolved risks

Localized parameter changes, particularly reductions in shock impedance, were observed when PFA was delivered near-exposed ICD coils. This occurred in a single case and was not reproducible, limiting definitive mechanistic conclusions. Importantly, these conditions represent a pre-specified worst-case experimental scenario rather than typical clinical practice.

Several explanations should be considered. Mechanical factors, including subtle catheter–lead interaction or transient displacement, may have influenced measurements. The event also occurred late in the procedure, and physiological or procedural factors may have contributed. Electroporation-induced changes in tissue conductivity may provide an additional explanation. Increased membrane permeability can transiently alter the electrical properties of myocardial tissue and blood,^21–25^ potentially modifying current pathways and affecting impedance measurements without causing structural device injury.

The observed case involved an integrated bipolar ICD lead, in which the defibrillation coil contributes to the sensing circuit. In such configurations, local electrical changes around the coil may have a greater impact on measured parameters. Experimental data indicate that metallic structures can distort the electric field distribution during PFA.^25–27^ Although this observation is hypothesis-generating, a case report of irreversible intracardiac defibrillator circuit damage has been described with systems near conductive elements,^20^ suggesting that cardiac devices may be affected if sufficient protective mechanisms are lacking.

In contrast, no macroscopic structural damage was observed in the present study, suggesting that transient electrical or mechanical factors are more likely explanations.

Overall, these findings support general electrical compatibility under typical conditions while indicating that atypical responses may occur when PFA is delivered in very close proximity to exposed conductive components. Interactions between PFA and CIED systems are therefore likely both distance- and platform-dependent.

### Leadless ventricular pacemakers and S-ICDs: structural advantages in the context of PFA

Leadless ventricular pacemakers and S-ICDs demonstrated robust tolerance to PFA in the present study. Despite extensive ablation exposure, sensing and therapy delivery remained stable. The absence of intracardiac conductive leads likely reduces susceptibility to both mechanical and electrical interactions, suggesting that extravascular systems may offer structural advantages as PFA expands to right-sided targets.

However, isolated reports have described transient pacing threshold elevation or loss of capture in atrial leadless pacing systems during monopolar PFA delivered in the same chamber,^28^ as well as undersensing in a dual-chamber leadless pacemaker (AVEIR DR^TM^) during PFA.^29^ Accordingly, caution remains warranted when PFA is delivered in close proximity to leadless devices.

### Implications for ventricular PFA and ICD lead function

Although ventricular PFA was not evaluated in the present study, its potential interaction with ICD lead function warrants consideration. Compared with atrial ablation, ventricular PFA involves thicker myocardium and more heterogeneous substrates, which may alter electric field distribution and require higher energy delivery.^30–32^ In addition, unipolar energy configurations, more likely to be used in ventricular applications, have been associated with the broader electric field distribution compared with bipolar configurations,^32^ potentially increasing interaction with adjacent conductive structures such as the right ventricular defibrillator coil. Accordingly, these effects cannot be inferred from the present findings and require dedicated investigation.

Additional PFA near intracardiac ventricular lead components would strengthen the ventricular lead safety assessment. However, direct ventricular PFA with the VARIPULSE catheter was not feasible in this swine model because its large single-shot design prevented safe, reproducible ventricular positioning and carried substantial ventricular arrhythmia risk. Nevertheless, RA/SVC workflows exposed insulated RV lead segments and, in dual-coil ICD systems, the SVC defibrillator coil to PFA. Across these exposures, ventricular sensing, pacing threshold, and pacing impedance remained stable; however, PFA near the SVC defibrillator coil was associated with shock impedance reduction in one animal, with uncertain mechanism and clinical significance. Therefore, the present findings provide limited acute data on insulated RV lead exposure during right-sided PFA but do not establish the safety of PFA delivery near-exposed defibrillator coils or intracardiac RV lead components. Dedicated ventricular PFA studies with pre-defined safety endpoints are warranted.

### Clinical implications

These findings suggest that PFA performed at an anatomical distance from transvenous lead systems is unlikely to cause critical device dysfunction under the conditions examined in this study. However, procedures involving the RA and SVC require particular attention, as transvenous leads frequently traverse the ablation field and catheter–lead interactions may occur. The observed complete atrial lead dislodgement and micro-dislodgement during SVC manipulation indicate that clinically relevant complications may occur through mechanical catheter–lead interaction, even without critical device malfunction. Localized parameter changes may also occur when PFA is delivered near-exposed conductive components such as ICD coils. Accordingly, careful catheter manipulation, spatial awareness between ablation electrodes and intracardiac leads, and peri-procedural device monitoring may be advisable. These findings should be viewed as hypothesis-generating evidence of context-dependent PFA–CIED interactions, rather than proof of universal device safety.

### Limitations

This study has several limitations. First, it was performed in an acute swine model with newly implanted leads and therefore did not reproduce chronic lead fixation, fibrotic encapsulation, or lead maturation in humans. Because device implantation, catheter manipulation, and PFA delivery were performed during the same session, the leads were more mechanically vulnerable than chronically implanted human CIED leads. The smaller swine SVC/RA anatomy may also have increased susceptibility to catheter–lead interaction. Thus, the observed lead dislodgements and parameter perturbations should be interpreted as indicators of acute procedural mechanical vulnerability under sensitive conditions, rather than direct estimates of clinical risk in chronically implanted leads.

Second, although lead position was confirmed fluoroscopically at implantation and reassessment, catheter manipulation was not continuously monitored or recorded throughout all RA/SVC manoeuvers. Therefore, the exact timing of lead displacement could not always be captured, and the relative contributions of mechanical manipulation and PFA energy delivery cannot be definitively separated.

Third, the sample size was limited and may not detect rare or device-specific interactions. Moreover, only a bipolar circular variable-loop PFA catheter system was evaluated. Because PFA effects depend on waveform, electrode geometry, energy configuration, and current pathway, these findings should not be extrapolated to other catheter designs or energy configurations, including lattice-tip, focal, spline-based, unipolar, or monopolar systems.

Fourth, ventricular myocardial PFA was not performed. Although RA/SVC ablation exposed insulated ventricular lead segments and SVC defibrillator coils in dual-coil ICD systems, direct ventricular ablation adjacent to RV lead tips, RV pacing electrodes, or RV defibrillator coils was not evaluated. Coronary sinus and conduction-system pacing leads, including left bundle branch area pacing leads, were also not evaluated.

Fifth, amiodarone was administered to reduce ventricular arrhythmia risk and may have suppressed PFA-related atrial or ventricular arrhythmia induction. Finally, the pulse generator was positioned in a cervical pocket ∼15 cm from the intracardiac ablation field, making generator–field interaction unlikely but not systematically evaluated. Because this was an acute study, delayed device effects, progressive parameter changes, and late-onset abnormalities could not be assessed.

## Conclusion

In this acute pre-clinical model, PFA was not associated with catastrophic CIED dysfunction under the specific experimental conditions of this study. Device–PFA interactions were strongly context-dependent, with right-sided procedures and proximity to conductive components representing higher-risk scenarios. These findings should be considered hypothesis-generating rather than definitive evidence of device safety and highlight the potential for context-dependent interaction risks. Careful catheter manipulation, spatial awareness, and appropriate device monitoring may be advisable during right-sided PFA procedures in patients with CIEDs. Further validation in chronic and clinical settings is warranted.

## Supplementary Material

oeag124_Supplementary_Data

## Data Availability

The data underlying this article are available from the corresponding author upon reasonable request.

## References

[1] Wojtaszczyk A, Caluori G, Pešl M, Melajova K, Stárek Z. Irreversible electroporation ablation for atrial fibrillation. J Cardiovasc Electrophysiol 2018;29:643–651. 10.1111/jce.13454 29399927

[2] Reddy VY, Koruth J, Jais P, Petru J, Timko F, Skalsky I, Hebeler R, Labrousse L, Barandon L, Kralovec S, Funosako M, Mannuva BB, Sediva L, Neuzil P. Ablation of atrial fibrillation with pulsed electric fields: an ultra-rapid, tissue-selective modality for cardiac ablation. JACC Clin Electrophysiol 2018;4:987–995. 10.1016/j.jacep.2018.04.005 30139499

[3] Reddy VY, Neuzil P, Koruth JS, Petru J, Funosako M, Cochet H, Sediva L, Chovanec M, Dukkipati SR, Jais P. Pulsed field ablation for pulmonary vein isolation in atrial fibrillation. J Am Coll Cardiol 2019;74:315–326. 10.1016/j.jacc.2019.04.021 31085321

[4] Koruth JS, Kuroki K, Iwasawa J, Enomoto Y, Viswanathan R, Brose R, Buck ED, Speltz M, Dukkipati SR, Reddy VY. Preclinical evaluation of pulsed field ablation: electrophysiological and histological assessment of thoracic vein isolation. Circ Arrhythm Electrophysiol 2019;12:e007781. 10.1161/CIRCEP.119.007781 PMC6924932 31826647

[5] Verma A, Haines DE, Boersma LVA, Sood N, Natale A, Marchlinski FE, Calkins H, Sanders P, Packer DL, Kuck K-H, Hindricks G, Onal B, Cerkvenik J, Tada H, DeLurgio DB. Pulsed field ablation for the treatment of atrial fibrillation: PULSED AF pivotal trial. Circulation 2023;147:1422–1432. 10.1161/CIRCULATIONAHA.123.063988 PMC10158608 36877118

[6] Reddy VY, Anic A, Koruth J, Petru J, Funasako M, Minami K, Breskovic T, Sikiric I, Dukkipati SR, Kawamura I, Neuzil P. Pulsed field ablation in patients with persistent atrial fibrillation. J Am Coll Cardiol 2020;76:1068–1080. 10.1016/j.jacc.2020.07.007 32854842

[7] Reddy VY, Calkins H, Mansour M, Wazni O, Di Biase L, Bahu M, Newton D, Liu CF, Sauer WH, Goyal S, Iyer V, Nair D, Athill C, Hussein A, Whalen P, Melby D, Natale A. Pulsed field ablation to treat paroxysmal atrial fibrillation: safety and effectiveness in the AdmIRE pivotal trial. Circulation 2024;150:1174–1186. 10.1161/CIRCULATIONAHA.124.070333 PMC11458102 39258362

[8] Reddy VY, Gerstenfeld EP, Natale A, Whang W, Cuoco FA, Patel C, Mountantonakis SE, Gibson DN, Harding JD, Ellis CR, Ellenbogen KA, DeLurgio DB, Osorio J, Achyutha AB, Schneider CW, Mugglin AS, Albrecht EM, Stein KM, Lehmann JW, Mansour M. Pulsed field or conventional thermal ablation for paroxysmal atrial fibrillation. N Engl J Med 2023;389:1660–1671. 10.1056/NEJMoa2307291 37634148

[9] Crossley GH, Poole JE, Rozner MA, Asirvatham SJ, Cheng A, Chung MK, Ferguson TB, Gallagher JD, Gold MR, Hoyt RH, Irefin S, Kusumoto FM, Moorman LP, Thompson A. The Heart Rhythm Society (HRS)/American Society of Anesthesiologists (ASA) expert consensus statement on the perioperative management of patients with implantable defibrillators, pacemakers and arrhythmia monitors. Heart Rhythm 2011;8:1114–1154. 10.1016/j.hrthm.2010.12.023 21722856

[10] Lennerz C, O'Connor M, Schaarschmidt C, Reents T, Bourier F, Telishevska M, Lengauer S, Popa M, Wimbauer K, Holmgren E, Thoma M, Spitzauer L, Bahlke F, Krafft H, Englert F, Knoll K, Friedrich L, Blazek P, Hessling G, Kolb C, Deisenhofer I, Kottmaier M. Pulsed field ablation in patients with cardiac implantable electronic devices: an ex vivo assessment of safety. J Interv Card Electrophysiol 2024 May 22. doi: 10.1007/s10840-024-01758-2. Online ahead of print.10.1007/s10840-024-01758-238775921

[11] Iqbal SUR, Kueffer T, Panakal A, Roten L, Reichlin T. Safety of pulsed field ablation using a circular catheter in a patient with a cardiac implantable electronic device. HeartRhythm Case Rep 2024;11:111–113. 10.1016/j.hrcr.2024.10.018 PMC11862152 40018322

[12] Liu X . Pulsed field ablation can be a source of electromagnetic interference with cardiac implantable electronic devices. HeartRhythm Case Rep 2024;10:858. 10.1016/j.hrcr.2024.08.020 PMC11628818 39664673

[13] Karakasis P, Antoniadis AP, Fragakis N. Pulsed field ablation in cardiac implantable electronic device patients: a welcome advance, yet questions remain. Europace 2025;27:euaf153. 10.1093/europace/euaf153 PMC12365749 40701548

[14] Lampert J, Koruth J, Miller MA, Reddy VY. A novel etiology for automatic mode switching and ventricular noise reversion alerts: pulsed field ablation. HeartRhythm Case Rep 2024;10:855–857. 10.1016/j.hrcr.2024.08.006 PMC11628844 39664661

[15] Abdelsayed K, Downey MC, Bahbah A, Mariam Tarek Desouki M, Witt D, Zishiri E, Sharkey S, Abdelhadi RH, Sengupta JD. Pulsed-field ablation and device-device procedural interactions with cardiac implantable electronic devices. Heart Rhythm O2 2025;6:2022–2024. 10.1016/j.hroo.2025.09.005 PMC12800789 41541715

[16] Nair DG, Gabrah K, Doty B, Nair G, Reddy VY. Cardiac implantable electronic device software reset and damage after pulsed field ablation using a monopolar lattice-tip catheter. Heart Rhythm 2025;23:1027–1031. 10.1016/j.hrthm.2025.09.027 41067477

[17] Malyar CV, Kovacs B, Mahmoodi BK, Haeberlin A, Reichlin T, Yap SC. Permanent damage to implantable cardioverter-defibrillators during left-sided septal ventricular tachycardia ablation using a lattice-tip catheter: a case series. Heart Rhythm 2026;23:1035–1039. 10.1016/j.hrthm.2026.01.028 41581847

[18] Sachdev M, Chung MK, Al-Khatib S, Liu C, Miller L, Shanker AJ; Heart Rhythm Society. Device Interaction Between Medtronic Sphere-9™ Ablation Catheter and Biotronik ICD/CRT-D Systems. 2026. https://www.hrsonline.org/resource/device-interactions-medtronic-biotronik/

[19] Campos-Villarreal D, Steiger NA, Coll M, Sauer WH. Visible arcing observed with pulsed field ablation applications delivered near a left atrial appendage occlusion device. Heart Rhythm 2025;22:e1300–e1301. 10.1016/j.hrthm.2025.07.055 40759236

[20] Takahashi K, Kuwahara T, Makita T, Arisuda Y, Kadono K, Oshio T. Irreversible intracardiac defibrillator circuit damage induced by pulsed-field ablation: mechanistic insights. HeartRhythm Case Rep 2026;12:344–349. 10.1016/j.hrcr.2025.12.012 PMC13005073 41869056

[21] Sel D, Lebar AM, Miklavčič D. Feasibility of employing model-based optimization of pulse amplitude and electrode distance for effective tumor electropermeabilization. IEEE Trans Biomed Eng 2007;54:773–781. 10.1109/TBME.2006.889196 17518273

[22] Pavlin M, Kandušer M, Reberšek M, Pucihar G, Hart FX, Magjarević R, Miklavcic D. Effect of cell electroporation on the conductivity of a cell suspension. Biophys J 2005;88:4378–4390. 10.1529/biophysj.104.048975 PMC1305665 15792975

[23] Corović S, Lacković I, Šuštarič P, Šuštar T, Rodič T, Miklavčič D. Modeling of electric field distribution in tissues during electroporation. Biomed Eng Online 2013;12:16. 10.1186/1475-925X-12-16 PMC3614452 23433433

[24] Garcia PA, Rossmeisl JH Jr, Rafael VD. Electrical conductivity changes during irreversible electroporation treatment of brain cancer. Annu Int Conf IEEE Eng Med Biol Soc 2011;2011:739–742. 10.1109/IEMBS.2011.6090168 22254416

[25] Hogenes AM, Meijerink MR, van der Laan LJW, Ichikawa T, van Gulik TM, van Leeuwen TG, Overduin CG, Stommel MWJ. Effect of irreversible electroporation parameters and the presence of a metal stent on the electric field line pattern. Sci Rep 2020;10:13517. 10.1038/s41598-020-70308-3 PMC7421881 32782339

[26] Wang Z, Liang M, Sun J, Zhang J, Li Y, Xu L, Han Y. Epicardial pulsed-field ablation: impact of electric field and heat distribution induced by coronary metallic stents. Front Cardiovasc Med 2024;11:1445424. 10.3389/fcvm.2024.1445424 PMC11391106 39267803

[27] Wang Z, Liang M, Sun J, Zhang J, Li Y, Zhang D, Han Y. Effects of electric field and heat distribution due to trends in metal stent diameter in epicardial pulsed field ablation. Sci Rep 2025;15:18719. 10.1038/s41598-025-01965-5 PMC12119961 40436943

[28] Díaz JO, Siddiqui U, Arenas CE. Acute atrial pacing threshold elevation in an auricular leadless pacemaker during monopolar pulsed field ablation of the right inferior pulmonary vein: a first-in-human case. J Cardiovasc Electrophysiol 2026;37:658–662. 10.1111/jce.70259 41536031

[29] Kikuchi Y, Nomura T, Yoshiyama K, Kumazawa D, Mizuno Y, Onodera K, Yamashita K. A case of near-zero fluoroscopy ablation using a circular catheter in a patient with a dual-chamber leadless pacemaker. HeartRhythm Case Rep 2025;11:1126–1130. 10.1016/j.hrcr.2025.08.005 PMC12666935 41333877

[30] Nakagawa H, Farshchi-Heydari S, Sugawara M, Ikeda A, Maffre J, Sharma T, Lam P, Govari A, Beeckler CT, Altmann A, Jackman WM, Franz MR, Spangler T, Hussein AA, Nakhla S, Santangeli P, Saliba WI, Wazni OM. Real-time prediction of irreversible lesion size during pulsed field ablation: prospective validation of a novel ablation Index based on contact force and number of applications in a swine beating heart model. Circ Arrhythm Electrophysiol 2025;18:e013911. 10.1161/CIRCEP.125.013911 PMC12711265 41328568

[31] Pérez JJ, González-Suárez A. How intramyocardial fat can alter the electric field distribution during Pulsed Field Ablation (PFA): qualitative findings from computer modeling. PLoS One 2023;18:e0287614. 10.1371/journal.pone.0287614 PMC10621855 37917621

[32] Kueffer T, Casoni D, Goepfert C, Beslac O, Parodi C, Ramirez D, Garrott K, Koop B, Coe S, Hagstrom N, Gibert G, Roten L, Haeberlin A, Reichlin T. Dose-dependent ventricular lesion formation using a novel large-area pulsed field ablation catheter: a preclinical feasibility study. Heart Rhythm 2025;22:2322–2330. 10.1016/j.hrthm.2025.02.017 39947454

