# Supplementary material for: Context-dependent interactions between variable-loop pulsed field ablation and cardiac implantable electronic devices: a controlled preclinical study
Source: Eur Heart J Open. 2026 Sep 11;6(5):oeag124. doi: 10.1093/ehjopen/oeag124 (PMC13564213; doi:10.1093/ehjopen/oeag124)
Supplement: Supplementary file 1 [file oeag124_supplementary_data.zip › Supplementary_legends.docx]

**Supplementary Table 1. Procedural Characteristics and Device Parameter Changes During PFA**

**Supplementary Figure 1. Intracardiac EGM during PFA in the PM device**

**Supplementary Figure 2. Intracardiac EGM during PFA in the Ventricular Leadless Pacemaker**
